# Thrombocytopenia induced by giant atrial thrombus in rheumatic valve disease

**DOI:** 10.1007/s12471-017-0984-1

**Published:** 2017-04-06

**Authors:** G. Caldentey, R. San Antonio, E. Flores-Umanzor, S. Vázquez

**Affiliations:** Hospital Clínic de Barcelona, Barcelona, Spain

**Fig. 1:**
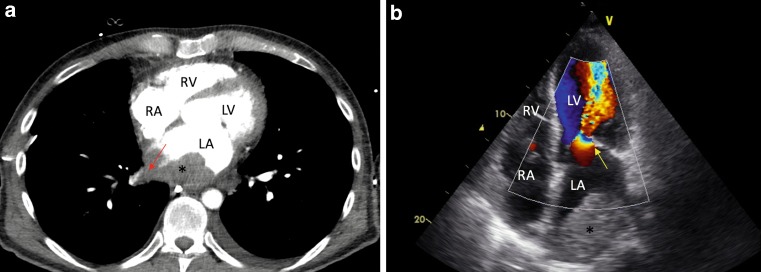
**a** Contrast thoracic CT scan showing a left atrial mass (*), with right inferior pulmonary vein infiltration (*red arrow*). **b** Apical four-chamber view from transthoracic echocardiography showing an enlarged left atrium with a giant left atrial thrombus (*). Rheumatic mitral valve with bileaflet restriction and commissural fusion resulting in severe stenosis (*yellow arrow*). *LA* left atrium, *LV* left ventricle, *RA* right atrium,* RV* right ventricle

A 48-year-old man with no previous medical history presented with congestion signs and rapid atrial fibrillation. There was evidence of thrombocytopenia (16,000/mcl) and moderate elevation of transaminases. An abdominal echography showed no liver alterations and no splenomegaly. A thoracic CT scan revealed a giant left atrial mass with pulmonary vein infiltration (Fig. 1a). Transthoracic echocardiogram confirmed this finding and showed rheumatic mitral valve stenosis (Fig. 1b). The patient underwent bioprosthetic valve implantation and removal of the giant thrombus. The platelet count progressively increased achieving normal levels one week after surgery.

Mitral stenosis increases blood stasis, representing a major risk factor for left atrial clot formation [1, 2]. Severe thrombocytopenia, in the absence of heparin treatment or major hepatic dysfunction, could be explained by ‘acute thrombosis-associated thrombocytopenia’ [3]. Hypothesis suggested that large fresh clots consume platelets on their surface, likely due to the exudation of thromboplastic substances. This process can be regarded as a form of disseminated intravascular coagulation [4, 5].
